# Clinical Pharmacology Aspects in Patients Treated with TNF Inhibitors During SARS-Cov-2 Pandemic

**DOI:** 10.34172/apb.2021.045

**Published:** 2020-10-20

**Authors:** Francesco Ferrara, Priscilla Santilli, Vilma D'Aiuto, Antonio Vitiello

**Affiliations:** Usl Umbria 1, Perugia, Italy.

**Keywords:** TNF inhibitors, SARS-Cov-2, Immunomodulating, Infection, Hyperinflammatory

## Abstract

In this period of global pandemic caused by SARS-Cov-2, it is of paramount importance to recognize all risk factors that may increase the likelihood of infection. In addition to the risk factors known as pre-existing diseases and old age, risk factors could be drug treatments for chronic diseases, such as immunomodulating drugs that can alter immune defences and response to infectious agents. Antibodies that inhibit tumor necrosis factor (TNF) such as adalimumab infliximab etanercept and golimumab have been used for over 20 years in severe cases of autoimmune diseases such as rheumatoid arthritis, inflammatory bowel disease or ankylosing spondylitis. Due to their mechanism of action they reduce inflammation and can stop the progression of the disease by inhibiting a key factor of inflammation such as TNF. In this article we want to examine the possible correlation between therapy with TNF inhibitors and the increased risk of SARS-CoV-2 infection, and the possible paradoxical therapeutic efficacy in patients with ongoing infection, especially in phase two and three. We express our opinion on this very complex and sensitive topic which is the subject of discussion among physicians and experts, based on current knowledge of the literature.

## Introduction

### SARS-Cov-2 and risk factors

In December 2019 an excessive number of cases of pneumonia caused by one coronavirus identified as SARS-CoV-2 occurred in China. This coronavirus has shown a rapid spread in China and also in other countries causing thousands of deaths. Based on the studies of these months, SARS-CoV-2 infection has been divided into three phases: phase 1, asymptomatic or slightly symptomatic incubation period that does not require hospitalization with or without detectable virus; phase 2, nonsevere symptomatic phase with the presence of the virus; phase 3, acute respiratory symptomatic period with high viral load and generalized hyperinflammatory state. Phase 3 is the most complicated and severe phase, and the generalized hyperinflammatory state can cause fatal lung injury and multi-organ dysfunction, this generalized inflammatory state to all tissues of the body is caused by a sudden release of circulating cytokines defined as “cytokinic storm”. Studies show that there is increasing evidence that those with pre-existing comorbidities, advanced age or compromised immune system are at higher risk of developing serious respiratory and viral disease infections. Risk factors may also include drug therapies, cancer patients on cancer therapy or patients with autoimmune diseases or transplantation such as rheumatoid arthritis and on immunosuppressive therapy are considered in this risk group. The current review focuses on the effects of tumor necrosis factor (TNF) inhibitors, indicated in autoimmune diseases, and their effect on host immune defences in the fight against SARS-Cov-2. We also examine their possible use during SARS-Cov-2 infection, particularly in stages two and three of infection. The purpose of this article is therefore twofold, it aims to identify whether TNF inhibitory drugs should be discontinued during this period of SARS-Cov-2 pandemic to avoid an increased risk of infection, and whether these drugs could potentially be useful in the fight against the hyperinflammatory state of phase two and three infection.^1-3^

## The patient undergoing treatment with TNF inhibitors

TNF inhibitors are indicated in several autoimmune diseases such as rheumatoid arthritis, ankylosing spondylitis, psoriatic arthritis, psoriasis, Crohn’s disease, ulcerative colitis and uveitis. These drugs selectively bind to TNF and neutralize its biological function. This mechanism of action is the basis of their therapeutic effectiveness in many of the diseases described above, but their use can also affect the body’s defenses against infections mainly of the respiratory tract. Furthermore, it must be considered that patients with autoimmune diseases are recognized as patients at high risk of infection due to the disease itself. TNF plays a critical role in infection control, in particular the release of TNF from the cells of the immune system, plays a critical role in defence against pathogenic organisms. TNF is an important pathway for the immune defense response against external pathogens and its inhibition may be a risk factor for a variety of infections. However, anti-TNF therapies may also have a regulatory role with potential beneficial effects on the immune system. Based on the current literature and meta-analyses studied, the risk data of patients receiving TNF-inhibitor therapy for infections are unclear and inconsistent. Several studies show that the risk of infection associated with anti-TNF therapy is higher in the first months of therapy and then decreases, also depending on the age of the patient. This variation in risk is probably explained by several factors, in particular one hypothesis could be that a persistent blockage of one cytokine pathway may lead to increased activation of the other immune pathways to compensate for the lack of TNF. The evidence therefore shows that there may be a slight increase in the risk of infection, as also reported in the reference CPRs. However, the evidence at this time does not recommend discontinuation of these drugs to virus-negative patients, but it is important to refer to the literature and the positions expressed by the relevant scientific societies. Paradoxically, however, the use of TNF inhibitors could be beneficial in stages two and three of the infection. In fact, in these stages of the infection the delicate inflammatory/immune balance is dysregulated, with a hyperactive inflammatory state, which can lead to a cytokinic storm responsible for lung lesions and multi-organ dysfunction that can cause death. Blockage of this cytokinic storm and mediators such as interleukin-1 (IL-1), IL-6 or TNF may be curative. Several studies are currently underway to test the effectiveness of cytokine blocking drugs such as IL-6 inhibitors, IL-1 and even TNF inhibitors. There is currently good evidence of the use of TNF inhibitors in stages two and three of infection, but data from trials are expected to confirm efficacy. It is likely that the use of multiple drugs in combination such as IL-6, IL-1 and TNF inhibitors may be more effective by blocking multiple pathways of inflammatory activation.^4-11^

## Conclusion

Due to their effectiveness and tolerability, anti-TNF-α agents are widely used for the treatment of many diseases. The purpose of this article is twofold, to identify whether TNF inhibitor drugs should be discontinued during this period of SARS-Cov-2 pandemic to avoid an increased risk of infection, and whether these drugs could potentially be useful in the fight against the hyperinflammatory state of phase two and three infection. From literature research there is currently no evidence to indicate that blocking TNF is harmful or may increase the risk of SARS-Cov-2 infection and it is not recommended to discontinue them, however we think that the use of these drugs could be curative in phases two and three of SARS-Cov-2 infection to block the hyperactive inflammatory state, but not in phase one, where the inflammatory and immune systems must fight and prevent viral replication. Ongoing clinical studies will give us the necessary evidence.

## Ethical Issues

The document does not conflict with ethical legislation.

## Conflict of Interest

We have no conflict of interest.
